# Gene flow analysis method, the D-statistic, is robust in a wide parameter space

**DOI:** 10.1186/s12859-017-2002-4

**Published:** 2018-01-08

**Authors:** Yichen Zheng, Axel Janke

**Affiliations:** Biodiversität und Klima Forschungszentrum, Senckenberg Gesellschaft für Naturforschung, 60325 Frankfurt, Germany

**Keywords:** Gene flow, The D-statistic, Sensitivity, Population size, Parameter space, Simulation

## Abstract

**Background:**

We evaluated the sensitivity of the D-statistic, a parsimony-like method widely used to detect gene flow between closely related species. This method has been applied to a variety of taxa with a wide range of divergence times. However, its parameter space and thus its applicability to a wide taxonomic range has not been systematically studied. Divergence time, population size, time of gene flow, distance of outgroup and number of loci were examined in a sensitivity analysis.

**Result:**

The sensitivity study shows that the primary determinant of the D-statistic is the relative population size, i.e. the population size scaled by the number of generations since divergence. This is consistent with the fact that the main confounding factor in gene flow detection is incomplete lineage sorting by diluting the signal. The sensitivity of the D-statistic is also affected by the direction of gene flow, size and number of loci. In addition, we examined the ability of the f-statistics, ${\widehat{f}}_G$ and ${\widehat{f}}_{hom}$, to estimate the fraction of a genome affected by gene flow; while these statistics are difficult to implement to practical questions in biology due to lack of knowledge of when the gene flow happened, they can be used to compare datasets with identical or similar demographic background.

**Conclusions:**

The D-statistic, as a method to detect gene flow, is robust against a wide range of genetic distances (divergence times) but it is sensitive to population size. The D-statistic should only be applied with critical reservation to taxa where population sizes are large relative to branch lengths in generations.

**Electronic supplementary material:**

The online version of this article (10.1186/s12859-017-2002-4) contains supplementary material, which is available to authorized users.

## Background

Traditional phylogenetic analyses that assume a bifurcating tree fails to model complicated evolutionary processes such as incomplete lineage sorting (ILS), gene flow, and horizontal gene transfer [1]. Gene flow, or introgression, refers to alleles from one species entering a different (and usually closely related) species through migration and hybridization. It is a violation of the assumption in traditional phylogenetics that speciation is a sudden event and no exchange of genetic information occurs thereafter. Incomplete lineage sorting refers to an occurrence where lineages of a certain locus fail to coalesce in the branch directly in the past of their population divergence, resulting in three or more un-coalesced lineages existing in a population [1, 2]. This can result in discordance between the genealogy of that locus (gene tree) and population split history (species tree). These factors caused phylogenetics to enter an era of multi-locus analysis and is facilitated by availability of whole-genome sequencing [3]. There are multiple methods designed to reconstruct a “species tree,” a tree that describes speciation processes as splitting of populations [4–7]. However, these methods still aim for a completely bifurcating tree. To fully resolve the complexity during speciation and divergence, one would need to treat “phylogenetic incongruence [as] a signal, rather than a problem” [8].

Analysis of gene flow must take ILS into account, because both processes generate gene trees that are incongruent with the species tree. Among the earliest methods to detect gene flow are a homoplasy-based analysis that finds taxa that are intermediate between putative parent species [9], and a gene tree comparison that identifies locus divergence younger than the species’ divergence [10]. Later methods can be generally separated into two categories: likelihood-based/Bayesian- and parsimony-based, using different interpretations of the coalescent models. Likelihood or Bayesian methods, such as Phylonet [11, 12] and CoalHMM [13] are based on a priori evolutionary models, and are applicable across a large range of conditions. However, their disadvantages often include excessive computation times and the need to estimate a large number of parameters and to specify priors that are difficult to obtain accurately, but which can crucially affect the outcome.

The D-statistic, also known as the ABBA-BABA statistic, is a useful and widely applied parsimony-like method for detecting gene flow despite the existence of ILS [14, 15]. This method is designed to be used on either one of two types of data: (1) sequence alignment where there is only one or a few samples per taxa, or (2) SNP data where the frequency of each allele in each population is known. This method compares the number of ABBA and BABA sites – parsimony-informative sites that support a different phylogeny than the species tree, and determine whether they are statistically equal in number. The two genealogies discordant with the species tree, ABBA and BABA are equally likely to be produced by ILS; therefore they should not differ in number if only ILS, but not gene flow is present. A significant difference between ABBA and BABA sites indicates that two non-sister species are more similar to each other than expected, which is interpreted as a signal of gene flow. The D-statistic has been used in numerous studies to detect gene flow between closely related species of bears [16], equids [17], butterflies [18] as well as hominids [14], and plants [19, 20], and even microbial pathogens [21].

**Fig. 1:**
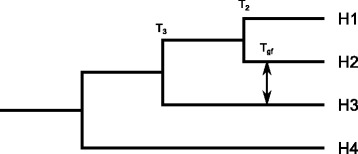
A four-taxon tree required to implement the D-statistic. The four taxa are designated as H1, H2, H3 and H4, with H4 serving as the outgroup. Gene flow between H2 and H3 (shown with arrows) or H1 and H3 can be detected with the D-statistic. T_3_, T_2_ and T_gf_denotes the time passed since each event.

The D-statistic (see Methods for formula) is used for a group of four taxa with an established phylogeny (Fig. 1) to detect gene flow between two ingroups that are not sister species (in this case, H2 and H3). The value of D is affected by a number of parameters; a) fraction of gene flow (f), b) divergence times, c) time of gene flow and d) population size. The “fraction of gene flow” refers to the fraction of recipient genome that descended from the donor population. The value of f cannot exceed 0.5, otherwise the source of gene flow will contribute more to the recipient’s genome than its lineage described in the species tree. As a result, the species tree would need to be changed to represent the lineages that provide the majority of the genome. Given the above parameters, the expected value of D is (formula from [15]):

$$ E(D)=\frac{3f\left({T}_3-{T}_{gf}\right)}{3f\left({T}_3-{T}_{gf}\right)+4N\left(1-f\right){\left(1-\frac{1}{2N}\right)}^{T_3-{T}_2}+4 Nf{\left(1-\frac{1}{2N}\right)}^{T_3-{T}_{gf}}} $$

Here f is the fraction of gene flow, N is the population size, T_3_ is the divergence time between the donor and recipient of gene flow, T_2_ is the divergence time between the recipient of gene flow and its sister species that have not received gene flow, and T_gf_ is the time of the gene flow event. All times are in units of generations. The expected value of D does not have a linear or mathematically simple relationship with the fraction of gene flow. Therefore, the calculation of f from D is impossible without knowing the divergence times, time of gene flow, and population size with high accuracy [22]. As a result, the D-statistic is often used as a qualitative measure where a significant D indicates presence of gene flow. Furthermore, the D-statistic can be highly susceptible to random variation in short sequences, making it unfit for detecting *which regions* have been affected by gene flow [22].

Durand et al. [15] proposed an alternative measure,${\widehat{f}}_G$ (see Methods for formula), which is expected to have a linear relationship with the actual fraction of gene flow, f, and is unaffected by population size. This is based on an assumption that a locus that underwent 100% gene flow will convert H2 into a member of the H3 population. Martin et al. [22] developed two additional estimators of f, ${\widehat{f}}_{Hom}$and${\widehat{f}}_d$. ${\widehat{f}}_{Hom}$ (see Materials and Methods for formula) uses the sequences of H3 as a control to determine how much of H2’s genome is affected by gene flow (see Materials and Methods), under an assumption that as the gene flow increases, H2 and H3 will be completely homogenized (which is only correct if the gene flow is extremely recent). ${\widehat{f}}_d$ compares H2 and H3 in a site-by-site basis and choose a “donor population” in which the derived allele has a higher frequency (therefore requiring population-level data), thus being able to explicitly model gene flow for both directions H2 - > H3 and H3 - > H2. Martin et al. [22] showed that both ${\widehat{f}}_G$ and ${\widehat{f}}_{Hom}$ have a high variance among loci and occasionally had a value above 1, indicating that they are subject to higher stochasticity; on the other hand, ${\widehat{f}}_d$ performs in a more stable way.

However, little is known about the parameter space in which the D- and f-statistics can be reliably used, which is of particular interest to biologists. The D-statistic is commonly used on species that diverged recently or have small genetic distances; it was originally developed to test hybridization between humans and Neanderthals, which diverged about 270,000–440,000 years ago (some 20,000 generations), and have a DNA sequence distance of 0.3% [14]. On the other hand, the method has been applied to species groups such as butterflies *Heliconius timareta* and *H. melpomene* [18], which were estimated to have diverged two million years ago with a DNA sequence distance of more than 1% [23]. This corresponds to 8 to 24 million generations, given a generation time estimate between one and three months [24, 25]. To date the maximal sequence divergence on which the D- and f-statistics have been applied is 4 to 5%, in mosquitoes of the genus *Anopheles* [26] and plants of the genus *Mimulus* [27]*.* It is still unknown if the D-statistic will be less effective on taxa that are highly diverged; an intuitive prediction would be the deterioration of the D-statistic’s effectiveness with increasingly divergent taxa, due to signals being overwhelmed by noise such as multiple substitutions and even saturation.

In the original simulation tests [15], the times of divergence and gene flow were not varied, and all polymorphic sites were independent without linkage. In the simulation tests by Martin et al. [22], the divergence times were strictly proportional to population size, not allowing variation of one without the other. The probability of two lineages (H1 and H2) coalescing on the branch leading to their divergence is determined by the ratio of branch length (in generations) and population size [28, 29]. If they fail to coalesce within the branch, a third lineage (H3) will appear in the population, leading to ILS, which produces two alternative gene trees that lead to ABBA and BABA sites at a same rate. The ratio of population size to divergence time, being a direct determinant of frequency of incongruent gene trees [1], is expected to have an effect on the sensitivity of the D- and f-statistics, i.e. how likely a gene flow event can be detected given that it exists. We predict that the D-statistic is less sensitive, and the f-statistics are less robust, in datasets with a higher population size relative to divergence time.

Therefore, we raise the question whether the effectiveness of the D- and f-statistics are affected by variation of divergence and gene flow time as well as population size, particularly when the ratio between population size and time scale is varied. In addition, we analyzed the statistical significance of the D-statistic instead of the statistic itself, particularly its sensitivity, because it is better suited as a qualitative measure. Finally, we will analyze the effect of gene flow direction and locus size on the statistical significance of the D-statistic, and the interaction between these variables (in particular, divergence times and gene flow direction). We are convinced that this will provide a valuable guide for future geneticists to better judge limits of incorrect interpretation of the D- and f-statistics as a method to detect and measure gene flow.

## Methods

### Definition of the D and f-statistics

According to the notions used by [15, 22] we review the parameters and their definitions used in the D and f- statistics for this study. Assume aligned or mapped DNA sequences are sampled from an asymmetric phylogeny of four taxa, (((H1, H2), H3), H4). *N*_*ABBA*_(*H*1, *H*2, *H*3, *H*4) is defined as the number of nucleotide sites in which H2 and H3 share an allele, while H1 and H4 share a different allele. Similarly, *N*_*BABA*_(*H*1, *H*2, *H*3, *H*4) is the number of nucleotide sites in which H1 and H3 share an allele, while H2 and H4 share a different allele. These numbers can refer to either one locus or the entire genome. The D-statistic is denoted as:

$$ D\left(H1,H2,H3,H4\right)=\frac{N_{ABBA}\left(H1,H2,H3,H4\right)-{N}_{BABA}\left(H1,H2,H3,H4\right)}{N_{ABBA}\left(H1,H2,H3,H4\right)+{N}_{BABA}\left(H1,H2,H3,H4\right)} $$

The numerator of this formula is represented by *S*(*H*1, *H*2, *H*3, *H*4).

In addition to the D-statistic, we examined two f-statistics that can be calculated without requiring the allele frequency in populations. These statistics, ${\widehat{f}}_G$ and ${\widehat{f}}_{hom}$, are estimators of f, the fraction of gene flow. While they utilize four taxa with the same tree as the D-statistic, ${\widehat{f}}_G$ has an additional requirement that at least two samples must be collected from the H3 population. The f-statistics are calculated as:

$$ {\widehat{f}}_G=\frac{S\left(H1,H2,H3,H4\right)}{S\left(H1,H3a,H3b,H4\right)} $$

$$ {\widehat{f}}_{hom}=\frac{S\left(H1,H2,H3,H4\right)}{S\left(H1,H3,H3,H4\right)} $$

H3a and H3b are two samples from the H3 lineage, assuming to be two unrelated individuals in the same population. The H3 used in the calculation of ${\widehat{f}}_G$ can be either H3a or H3b. For ${\widehat{f}}_{hom}$, H3 is used twice in the denominator; *N*_*BABA*_(*H*1, *H*3, *H*3, *H*4) is always zero, because H3 cannot be different from itself, so *S*(*H*1, *H*3, *H*3, *H*4) is identical to *N*_*ABBA*_(*H*1, *H*3, *H*3, *H*4), i.e., alleles shared by H1 and H4 but not by H3.

Tests of significance for the D- and f-statistics were done with a jackknife method, in which 5 Mb blocks were removed one at a time to estimate a standard error that is approximately normally distributed [30, 31].

### Simulating of species trees, gene trees and DNA sequences

**Fig. 2:**
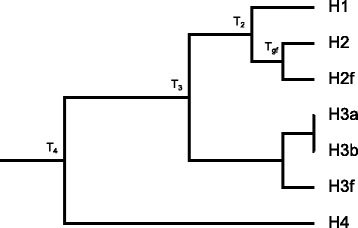
The species tree used for the coalescent-based gene tree simulation. T_gf_, T_2_, T_3_ and T_4_ are respectively divergence or gene flow times of the corresponding event, measured in the unit of generations. H3a and H3b represent two independent samples from the same H3 population. H2f represents an introgressed lineage originating from the H2 population, and similarly H3f represents an introgressed lineage originating from the H3 population

We used coalescence models to simulate gene trees from species trees, in order to take in account ILS in addition of gene flow. A species tree with fixed topology (Fig. 2) was used as the basis of the simulation, in which T_gf_, T_2_, T_3_ and T_4_ are independent variables we control in input. Of note is that H3a and H3b represent two samples from the same population, used to calculate ${\widehat{f}}_G$. H2f and H3f are used as lineages introduced by gene flow, that *originates* from H2 and H3 respectively. The parameters were set according to Table 1 (Scheme 1), producing 27 species trees with different branch lengths. Note that both branch length and population size were scaled with the reciprocal of substitution rate, 1/μ, so that the results would be applicable to organisms with a wide range of substitution rates. Along a branch with the length T = k × 1/μ generations, k substitutions per nucleotide are expected.

**Table 1:** Variables and constant parameters used in the study

Variable	Scheme 1: analysis of branch lengths and population	Scheme 2: analysis of outgroup distance	Scheme 3: analysis of number and size of loci	Scheme 4: analysis of diploid data
Divergence (T_3_)	0.001, 0.01 or 0.1 × 1/μ Generations	0.001 or 0.01 × 1/μ Generations	0.001 or 0.01 × 1/μ Generations	0.001 or 0.01 × 1/μ Generations
T_gf_/T_2_	0.25, 0.5 or 0.75	*0.5*	*0.5*	One of these combinations: 0.25 and 0.1; 0.5 and 0.5; or 0.75 and 0.9.
T_2_/T_3_	0.1, 0.5 or 0.9	*0.5*	*0.5*	
T_4_/T_3_	*2*	1.5, 2, 5, 10 or 20	*2*	*2*
Population size	0.2, 0.5, 1, 2 or 5 T_3_	0.2, 0.5, 1, 2 or 5 T_3_	0.2, 0.5, 1, 2 or 5 T_3_	0.2, 1, or 5 T_3_
Number of loci	*50,000*	*50,000*	2000, 5000, 10,000, 20,000, 50,000 or 100,000	*50,000*
Replicates	*3*	*3*	*3*	*3*
Total datasets	405	150	180	54

Datasets were simulated in three schemes, focusing on different parameters. Parameters not varied in that scheme are in *italics*

SimPhy [32] was used to simulate gene trees from species trees, using a coalescence-based Wright-Fisher model [33, 34]. The population size, Ne, is constant throughout the tree and proportional to divergence level (Table 1, Scheme 1). Gene trees were produced from each species tree; 15 sets of 50,000 gene trees were produced, which include three replicates for each of the five population sizes. In each gene tree, a sample of each lineage (H1, H2, H2f, H3a, H3b, H3f and H4) was taken and the divergence times between *samples* were simulated as constrained by the species tree, i.e. divergence times between *populations*. The resulting gene tree may have a different topology than the species tree. We denote the ratio Ne/T_3_ as the “relative population size.” A total of 135 parameter combinations and 405 datasets were generated. All other parameters were set to default.

The branch lengths in the simulated gene trees were then converted from units of generations to units of substitutions per nucleotide, during which the parameter 1/μ was cancelled out. The program INDELible [35] was used to simulate non-coding DNA sequences from gene trees. A 20-kb-long locus was simulated from each gene tree. The sequence evolution model was HKY with a transition/transversion ratio of 3.6 [36, 37], gamma distribution of substitution rate with shape factor α = 1, and a GC content of 40%. Each of the 135 parameter combinations produced 50,000 unlinked loci, with a total size of 1Gb. A typical mammalian genome is 3Gb and contains about a half repeat sequences; thus, 1Gb is close to the size of a mammalian genome alignment with repeats and difficult-to-map regions (such as centromeres and telomeres) excluded.

ABBA and BABA site counts for D, the ${\widehat{f}}_G$ and ${\widehat{f}}_{hom}$ statistics were calculated in each locus, under three alternative situations: (1) under no gene flow, H1, H2, H3a and H4 are used as the four sampled sequences, and H3a and H3b are used as two samples of H3 to calculate ${\widehat{f}}_G$; (2) under gene flow from H3 to H2. Here H1, H3f, H3a and H4 are used as the four sampled sequences, and H3a and H3b are used as two samples of H3 to calculate ${\widehat{f}}_G$; (3) under gene flow from H2 to H3, H1, H2, H2f and H4 are used as the four sampled sequences. Calculation of ${\widehat{f}}_G$ in (3), as it requires sampling two individuals in the gene flow recipient, is deemed to be beyond the scope of this study. The reason is that when two samples of H3 (recipient of gene flow) are taken, it is possible that only one sample is introgressed in a certain locus; however this possibility is dependent on whether the introgressed allele is fixed, which requires a more complicated coalescence model.

Hereafter, an “introgression test” refers to the following procedures: given a fraction of gene flow of a certain direction, f (0 ≤ f ≤ 0.5), in a 1Gb dataset, 50,000 × f loci are randomly chosen to be under gene flow, while the other 50,000 × (1-f) loci are not under gene flow. Using this combination, the D, ${\widehat{f}}_G$ and ${\widehat{f}}_{hom}$statistics are calculated using the formulae detailed above and tested using the jackknife method, where every 250 loci (5Mbp) are used as one block [14]. A test is significant if the resulting Z score (the value of D-statistic divided by its standard error) is above 3, a value chosen for strong significance based on [14, 38] corresponding to *p* < 0.0013. The Z score of the D, ${\widehat{f}}_G$ and ${\widehat{f}}_{hom}$ statistics are calculated separately, therefore, their significance are also determined separate from each other. In summary, an introgression test is a test for the D- and f-statistics and their significance, given the fraction of gene flow, f, and the dataset.

### Sensitivity test

A sensitivity test is an analysis on parameters that would cause false negatives in a test. In our case, the sensitivity test is a power analysis; determining the power of the D-statistic to detect gene flow. Sensitivity tests were conducted in two steps. In the first step, f values of 0, 0.001, 0.002, …, 0.009, 0.01, 0.015, 0.02, 0.03, …, 0.09, 0.1, 0.15, 0.2, 0.3, 0.4, and 0.5 (hereafter called the “basic f list”) were used for introgression tests. Each f value other than 0 was tested 3 times. The smallest f for which all 3 times tested positive was denoted f_0_; the number two places before f_0_ in the “basic f list” was denoted f_min_ (if f_0_ = 0.001 or 0.002, f_min_ = 0.001), and the number immediately after f_0_ was denoted f_max_ (if f_0_ = 0.5, f_max_ = 0.5). In the second step, f values between f_min_ and f_max_ were tested with an interval of 0.001. Each f value was tested 500 times. Using a logistic regression, the smallest f that have an 80% probability to produce a significant result was used as the threshold value to indicate sensitivity, as standard for power analyses [39]. This threshold is called MF80, (Minimal Fraction for 80% significance), and lower MF80 indicates better sensitivity. If the predicted probability of the D-statistic being significant is still less than 80% when f = 0.5, the D-statistic is not usable in this dataset. In this situation, MF80 is set as 0.501 for the downstream statistical analysis rather than treating it as missing data, so that we can make use of the knowledge that the D-statistic is extremely insensitive in this dataset. It will only cause underestimation of the correlations between sensitivity and parameters as the true MF80 (had we allow f > 0.5) will be at least 0.501.

The ${\widehat{f}}_G$ and ${\widehat{f}}_{hom}$statistics were linearly regressed with the input f using the data from the entire “basic f list”; the slope of this regression is used as estimate of ${\widehat{f}}_G$ /f and ${\widehat{f}}_{hom}$/f.

### Analyzing the effect of outgroup distance

In this section, we studied how the genetic distance between outgroup (H4) and ingroups (H1-H3) affect the sensitivity of the D- and f-statistics, given an otherwise identical species tree. The variables used in this section are described in Table 1 (Scheme 2). Of note is that the highest level of divergence is not included because it is least realistic, and the T_4_/T_3_ ratio is the main variable under study. From each parameter combination, three replicates each of 50,000 gene trees were simulated, and from each gene tree, 20 kb of non-coding DNA sequences were simulated, using the same method as the previous section. A total of 150 datasets were produced. Analysis of sensitivity of the D- and f-statistics are also conducted using the same methods as the previous section.

### Analyzing the effect of number and size of independent loci

In this section, we studied the impact on the D- and f-statistics by the number of independent loci, given the same species tree and total sequence length. The variables used in this sections are described in Table 1 (Scheme 3). Of note is that the highest level of divergence is removed, and the locus number is the main variable under study. Under a constant total sequence length of 1Gb, the lengths of each locus under each value are 500 kb, 200 kb, 100 kb, 50 kb, 20 kb and 10 kb. From each parameter combination, three replicate datasets were simulated, producing 180 datasets in total. Analysis of the D- and f-statistics were conducted using the same methods as in previous sections.

### Robustness of f-statistics

This section describes an analysis on the robustness of the f-statistics against random variation caused by locus sampling. We used data from 18 parameter combinations in Simulation Scheme 1: T_3_ = 1 × 10^4^ or 1 × 10^5^ Generations; Population size = 0.2, 1 or 5 T_3_; T_gf_/T_2_ and T_2_/T_3_ are one of these combinations: 0.25 and 0.1, 0.5 and 0.5, or 0.75 and 0.9.

The f-statistics we examined are ${\widehat{f}}_G$ and ${\widehat{f}}_{hom}$ in H3 - > H2 gene flow, and ${\widehat{f}}_{hom}$ in H2 - > H3 gene flow. For each real f value on the “basic f list” (see above section “Sensitivity Test”) we estimated 500 replicate sets of the f-statistics. In each replicate, 50,000 loci are randomly selected from the combined pool of 150,000 loci of the three replicates of that parameter combination (Table 1); within which, f × 50,000 of them are under gene flow and (1-f) × 50,000 are not under gene flow. The f-statistics were calculated and their confidence intervals were determined as (statistic ±2× standard deviation) [15]. In a small number of replications, the jackknife variance of ${\widehat{f}}_G$ was calculated as negative (the variance is based on a weighted measure where the weight of a jackknife block is based on the denominator of the f-statistic, which can be negative in some blocks for ${\widehat{f}}_G$, because the formula includes a subtraction); in these cases the confidence intervals were treated as missing data.

Pairwise comparisons were conducted in these procedures:

Let i and j be real f values from the “basic f list”, where i ≤ j. Compare each of the 500 replicate ${\widehat{f}}_G$ values where the real f is i (${\widehat{f}}_G(i)$), and each of the 500 replicate ${\widehat{f}}_G$ values where the real f is j (${\widehat{f}}_G(j)$); in the 500 × 500 = 250,000 comparisons, record the proportion of comparisons where ${\widehat{f}}_G(i)$ is *numerically* smaller than ${\widehat{f}}_G(j)$, and where ${\widehat{f}}_G(i)$ is *significantly* smaller than ${\widehat{f}}_G(j)$; in the case where i = j, record the proportion of comparisons where ${\widehat{f}}_G(i)$ is *not significantly different* from ${\widehat{f}}_G(j)$. Significant difference is defined by non-overlapping confidence intervals. The same procedures were also used to compare ${\widehat{f}}_{hom}$ from gene flow of both directions. The recorded proportions are estimates of the probability that the difference between real f values (or lack thereof) were correctly identified using the f-statistic.

### Diploid data

To study whether our findings are applicable to diploid data, we simulated additional datasets. The variables used in this section are described in Table 1 (Scheme 4), and the 18 parameter combinations are a subset of the ones from Scheme 1: T_3_ = 1 × 10^4^ or 1 × 10^5^ Generations; Population size = 0.2, 1 or 5 T_3_; T_gf_/T_2_ and T_2_/T_3_ are one of these combinations: 0.25 and 0.1, 0.5 and 0.5, or 0.75 and 0.9. Gene trees and sequences were simulated using the same procedures as in previous schemes, except that we specified two sequences were sampled from each taxon. One combination of parameters (T_3_ = 1 × 10^5^ Generations, Population size = 5 T_3_, T_gf_/T_2_ = 0.5, T_2_/T_3_ = 0.5) had an additional replication simulated, because one of the original replications resulted in a false positive (Z > 3 when no gene flow is present) and was discarded.

Analysis of sensitivity of the D-statistic were conducted using similar methods as the previous sections with special consideration taken for diploid data. During the introgression tests, two methods were used to draw the loci under gene flow for the recipient taxon. In the “same loci” method, the same 50,000 × f loci are randomly chosen to be under gene flow for both genome copies; in the “random loci” method, two independent sets of 50,000 × f loci (allowing overlap) are chosen for the two genome copies. Sites that are heterozygous in any analyzed taxon were excluded from the ABBA and BABA site counts.

## Results

### Sensitivity of the D-statistic in relation with divergence time, branch lengths, population size and direction of gene flow

**Fig. 3:**
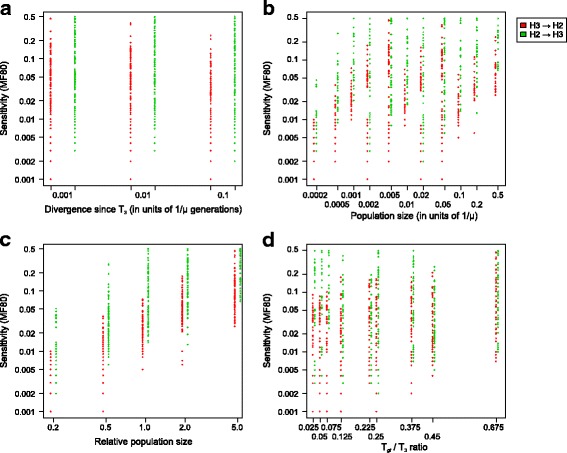
Sensitivity and input parameters. The relationship of sensitivity as measured with MF80, the minimal fraction of gene flow that produces over 80% significant D-statistics, and various input parameters: **a** Divergence, measured in generations between H3’s divergence and current time (T_3_); **b** Population size; **c** Relative population size, the ratio of population size and divergence generations; **d** Relative time of gene flow, the ratio of time of gene flow (T_gf_) and T_3_. Red points represent gene flow from H3 to H2, and green points represent gene flow from H2 to H3; the colors are slightly offset on the x-axis to ease reading

Sensitivity of the D-statistic is described with the minimal fraction of gene flow to have an over 80% probability producing a significant (Z > 3) test result. We call this value **MF80** (Minimal Fraction for 80% significance), and lower MF80 indicates better sensitivity. Figure 3 shows the relationship between four parameters and MF80. Our simulations show that, counterintuitively, MF80 has only a marginal negative correlation with divergence time (*r* = −0.146, *p* = 0.003; for *log* MF80 and *log* divergence time, *r* = −0.105, *p* = 0.034), which indicates a (slightly) better sensitivity in high divergence datasets. MF80 does not change markedly even with large divergences (sequence differences) (Fig. 3a), where H1/H2 and H3 have a genetic distance of over 20%. For comparison, mouse and rat have a sequence difference of 15–17% [40].

On the other hand, MF80 is affected by the population size (Fig. 3b, *r* = 0.151, *p* = 0.002), indicating better sensitivity with small populations. The correlation between *log* population size and *log* MF80 is stronger (*r* = 0.361, *p* < 0.0001); this is because population sizes were varied on a logarithmic scale, making the numbers crowd on the lower side when not log-transformed. The strongest signal, however, occurs when we compare MF80 with *relative* population size (Fig. 3c). Relative population size is defined as the ratio of population size and T_3_, which is number of generations passed since H1, H2 and H3 split in the species tree. For example, human and Neanderthal have a divergence time of 20,000 generations and an effective population size of about 10,000, so the relative population size is estimated as 10,000/20,000 = 0.5 [14]. The correlation between MF80 and relative population is *r* = 0.693 (*p* < 0.0001), and increases to *r* = 0.890 (p < 0.0001) if both are logarithmically transformed. Within each divergence category (0.001, 0.01 or 0.1 × 1/μ Generations), the pattern of correlation is same as for the entire combined dataset.

**Fig. 4:**
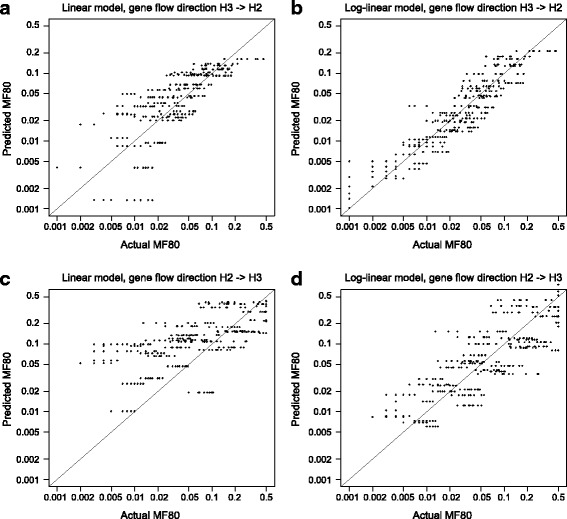
Comparison of measured and predicted sensitivity. Comparison between sensitivity as measured with MF80 measured from our analysis and MF80 predicted with a linear (**a**, **c**) or log-linear (**b**, **d**) model based on the relative population size and T_gf_/T_3_ ratio. **a**, **b** Direction of gene flow is H3 - > H2; **c**, **d** Direction of gene flow is H2 - > H3. The sloped line indicates when the measured and predicted MF80 are equal

Finally, there is a weak correlation between the T_gf_/T_3_ ratio and MF80 (Fig. 3d, *r* = 0.371, *p* < 0.0001; with log MF80, *r* = 0.349, *p* < 0.0001), indicating that gene flow events that are more recent are easier to detect. From the correlation analyses, it can be concluded that the sensitivity of the D-statistic is primarily determined by relative population size, and secondly determined by time of gene flow; indeed, these two variables can largely predict the output MF80 under a simple linear (Fig. 4a) or log-linear (Fig. 4b) model, with the latter being more accurate.

**Fig. 5:**
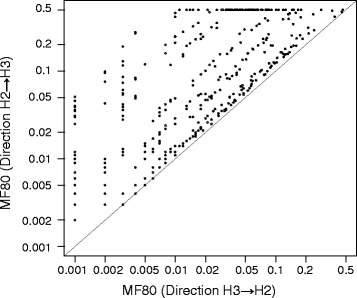
Comparison between sensitivity as measured with MF80 from two gene flow directions. Each point represents one of 405 datasets. The sloped line indicates where the MF80 from two directions are equal; all dots are on or above the line, implying that MF80 from H2 - > H3 gene flow is never lower than MF80 from H3 - > H2 gene flow

Gene flow from H2 to H3 was also simulated with the same methods, and the D-statistic’s sensitivities were measured as MF80 in all datasets. Regardless of divergence, population size or relative time of gene flow, the D-statistic is less or at most equally sensitive compared to gene flow from H3 to H2 (Fig. 5).

Correlations between input parameters to MF80 on the H2- > H3 direction were calculated with the same methods. Similar to the H3- > H2 direction, MF80 is not affected by the divergence (Fig. 3a, *r* = −0.098, *p* = 0.048; if both log transformed, *r* = −0.090, *p* = 0.070), weakly by absolute population size (Fig. 3b, *r* = 0.239, *p* < 0.0001; if both log transformed, *r* = 0.342, *p* < 0.0001), but strongly by relative population size (Fig. 3c, *r* = 0.826, *p* < 0.0001; if both log transformed, *r* = −0.090, *p* < 0.0001). However, the correlation between MF80 and the T_gf_/T_3_ ratio is *r* = −0.234 (Fig. 3d, *p* < 0.0001), meaning that younger gene flow events are more difficult to detect than older ones, a counterintuitive finding. The correlation becomes weaker if log(MF80) is used instead (*r* = −0.130, *p* = 0.009). Further investigation (Additional file 1) showed that MF80 is positively correlated with T_gf_/T_2_ ratio (*r* = 0.235, *p* < 0.0001), but strongly and negatively correlated with T_2_/T_3_ ratio (*r* = −0.440, *p* < 0.0001). This pattern is not found in the H3 - > H2 direction. When H1 and H2 diverged later (relative to H3 divergence time), i.e., T_2_/T_3_ ratio is lower, there are more shared alleles between H1 and (un-introgressed) H2. Under H3 - > H2 gene flow, these shared alleles become different, producing more ABBA sites. Under H2 - > H3 gene flow, on the other hand, these shared alleles become shared by all H1, H2, H3, producing BBBA patterns, and thus not counted. The ability of MF80 prediction by relative population size and T_gf_/T_3_ ratio is weaker than the H3 - > H2 direction (Fig. 4c, d).

For detailed MF80 on both directions for each dataset, see Additional file 2.

### Sensitivity of the D-statistic in relation with the distance of outgroup and number of loci

**Fig. 6:**
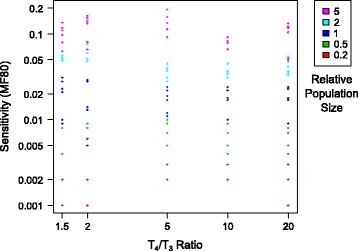
Sensitivity and distance of outgroup. The relationship between T_4_/T_3_ ratio (x-axis), and sensitivity as measured with MF80 (y-axis). A higher T_4_/T_3_ ratio indicates that the outgroup is more distant to the ingroups, relative to the distance among the ingroups. Colors represent results from analyses for different relative population sizes, with red being the smallest and purple the largest. The analyses show that MF80 is positively and strongly correlated to the relative population size, while MF80 is not notably affected by the T_4_/T_3_ ratio, either as a whole or within each relative population size

An intuitive expectation would be that the test’s sensitivity decreases when the outgroup (H4) is more distant, as a distant outgroup reduces the information quality and amount. Here we tested the effect of the outgroup distance, described by T_4_/T_3_ ratio, in a range from 1.5 to 20. In comparison, the ratio is about 7.9 times in the earliest usage of the D-stat where H3 is Neanderthal and H4 is chimpanzee ([14]; human-Neanderthal mean sequence divergence estimated as 825,000 years and human-chimpanzee as 6.5 million years). Figure 6 shows the sensitivity of the D-statistic, measured with MF80, under different T_4_/T_3_ ratio (x-axis) and relative population size (color). It is evident that MF80 is primarily determined by relative population size, but it is unaffected by the T_4_/T_3_ ratio. The correlation between T_4_/T_3_ ratio and MF80 is calculated to be *r* = −0.078 (*p* = 0.342), or *r* = −0.021 (*p* = 0.795) if log transformed. However, the interaction between T_4_/T_3_ ratio and relative population size is significant (*p* = 0.005). Therefore, we showed that the D-statistic is robust regarding the genetic distance between ingroups and the outgroup.

**Fig. 7:**
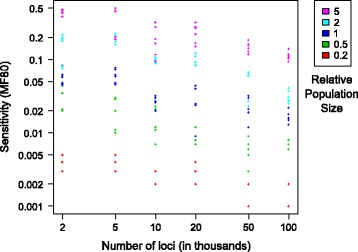
Sensitivity and number of independent loci. The relationship between the number of independent loci in each 1Gb dataset (x-axis), and sensitivity as measured with MF80 (y-axis). Colors represent results from analyses for different relative population sizes, with red being the smallest and purple the largest; MF80 is positively and strongly correlated with relative population size. MF80 is also correlated with number of loci, with a larger number of loci (thus smaller loci) resulting in lower MF80. The correlation between loci number and MF80 is weaker than the correlation between relative population size and MF80

In each of the above datasets, 1Gb of DNA sequences were simulated as 50,000 unlinked loci each of 20 kb. Here we analyzed the effect of locus number and size on sensitivity under a constant total sequence length of 1Gb. Figure 7 shows the sensitivity of the D-statistic, measured with MF80, for different numbers of loci (x-axis) and different relative population sizes (color). While the effect of the number of loci is not as strong as that of the relative population size, there is a trend that MF80 becomes smaller (more sensitive) in datasets with shorter sequences of each locus, but increasing number of loci. The correlation between locus number and MF80 is *r* = −0.273 (*p* = 0.0002), or *r* = −0.297 (*p* < 0.0001) when both are log transformed. The interaction between locus number and relative population size is not significant (*p* = 0.534) when both are transformed.

### The D-statistic when no gene flow is present

**Fig. 8:**
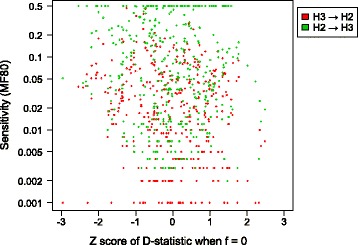
Z-score for the D-statistic under no gene flow. The relationship between the Z-score of the D-statistics under f = 0 (x-axis), and sensitivity as measured with MF80 (y-axis). Red points represent gene flow from H3 to H2, and green points represent gene flow from H2 to H3. The Z-score of the D-statistics under f = 0 is expected to be zero; any deviation is caused by random sampling error of loci (noise). There is a weak correlation between MF80 and Z-score of the D-statistics under f = 0, indicating that measured sensitivity is slightly influenced by sampling error of loci

One potential source of error in this study comes from the difference between ABBA and BABA site numbers even when no gene flow is occurring, due to the sampling error during gene tree and sequence simulation. For example, one would expect the MF80 to be underestimated if the zero-f dataset has a positive D-statistic, or vice versa. We used the Z-score of the D-statistic when f = 0 as an indicator of such bias. None of the 405 datasets have a significant Z-score (|Z| > 3) when f = 0 (which would constitute a false positive). This Z-score is significantly correlated with MF80 (Fig. 8) in the H3- > H2 direction, the correlation is *r* = −0.143 (*p* = 0.004), but not in the H2- > H3 direction, where *r* = −0.089 (*p* = 0.072). This indicates that the sensitivity is indeed affected by random sampling error, albeit only weakly so. However, we argue that this random noise is canceled out when all 135 datasets are used and it does not bias our general findings. The absolute value Z-score when f = 0 is not correlated with most input parameters (*p* = 0.926 for divergence, *p* = 0.076 for relative population size, and *p* = 0.056 for T_gf_/T_3_ ratio). For the individual Z-scores when f = 0 in each dataset, see Additional file 2.

### Usage and robustness of the f-statistics

In addition to the D-statistic, we tested the ${\widehat{f}}_G$ and ${\widehat{f}}_{hom}$statistics, which are estimates of f, the fraction of genome affected by the gene flow event; they were proposed because the D-statistic is qualitative and cannot be used to estimate how strong the gene flow is. In each of the 405 datasets, ${\widehat{f}}_G$ and ${\widehat{f}}_{hom}$are both linearly correlated to f where the gene flow direction is H3- > H2, with correlation coefficient r no smaller than 0.98 in any dataset. The ratios ${\widehat{f}}_G$ /f and ${\widehat{f}}_{hom}$/f are calculated with linear regression models; the estimated parameters of the models can be found in Additional file 2. As expected from [15], ${\widehat{f}}_G$ /f is roughly equal to $1-\frac{T_{GF}}{T_3}$. On the other hand, the ratio ${\widehat{f}}_{hom}$/f can be most closely estimated as $\left(1-\frac{T_{GF}}{T_3}\right)/\left(1+\frac{N_e}{T_3}\right)$ (See Additional file 2 for the predictors’ precision).

The intercept of the linear regression between ${\widehat{f}}_G$ (or ${\widehat{f}}_{hom}$) and f indicates an error, where the f-statistics are non-zero even without actual gene flow. In some datasets with low to medium divergence and large population sizes, $\left|{\widehat{f}}_G\ \right|$ can be above 0.05 even when f = 0, meaning that there will be a false positive of gene flow if it is used solely as a predictor of f. All 17 datasets where $\left|{\widehat{f}}_G\ \right|>0.03$ have divergence of 0.001 or 0.01 × 1/μ generations and a relative population size of 5. On the other hand, $\left|{\widehat{f}}_{hom}\ \right|$when f = 0 does not exceed 0.01 in all datasets, indicating that it is more robust against false positives compared to ${\widehat{f}}_G$.

Significance of ${\widehat{f}}_{hom}$can be tested in a similar way to the D-statistic, using jackknife subsampling. Indeed, the sensitivity of ${\widehat{f}}_{hom}$is almost identical to D; the MF80, minimal fraction of gene flow for 80% chance of significance (Z ≥ 3), are equal or close to equal in all datasets. On the other hand, ${\widehat{f}}_G$ is much more difficult to evaluate statistically. The main reason is that the jackknife makes use of the denominator in each block to determine the weight of each block in the entire dataset; the denominator of ${\widehat{f}}_G$ is the difference between two non-zero site counts, which can be negative or even zero, rendering the jackknife algorithm inapplicable.

${\widehat{f}}_G$ under H2 - > H3 gene flow was not calculated, because our model cannot predict whether the same introgressed loci are fixed for multiple samples in the recipient population. For most datasets, ${\widehat{f}}_{hom}$is linearly correlated with f, similar to the H3 - > H2 direction. However, the correlation is very weak in datasets with low T_2_/T_3_ ratio (very recent divergence between H1 and H2) and high relative population size, indicating that f cannot be predicted with ${\widehat{f}}_{hom}$even if all parameters are known. The slope of linear regression, ${\widehat{f}}_{hom}$/f can be estimated as $\left(\frac{T_2}{T_3}-\frac{T_{GF}}{T_3}\right)/\left(1+\frac{N_e}{T_3}\right)$ (See Additional file 2 for the predictor’s precision). This ratio is always smaller than what it could be if the direction of gene flow is H3 - > H2; the difference is stronger when T_2_/T_3_ ratio is low.

**Fig. 9:**
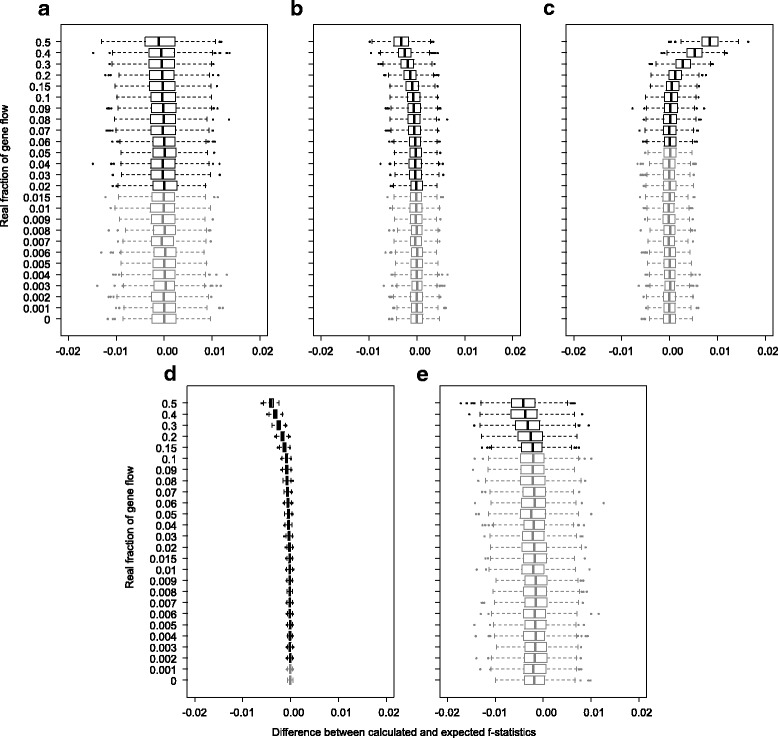
The difference between the estimated and expected f-statistics. For each scenario and each real f value, 500 bootstrap replicates were calculated. Gray boxes indicate real f values below the sensitivity of the D-statistic in the same scenario (mean of three replicates). In all graphs, the divergence time T_3_ = 1 × 10^5^ generations, T_GF_ = 0.5 T_2_ = 0.25 T_3_. **a** N_e_ = T_3_; ${\widehat{f}}_G$, gene flow direction H3 - > H2; **b** N_e_ = T_3_; ${\widehat{f}}_{hom}$, gene flow direction H3 - > H2; **c** N_e_ = T_3_; or ${\widehat{f}}_{hom}$, gene flow direction H2 - > H3; **d** N_e_ = 0.2 T_3_; data for ${\widehat{f}}_{hom}$, gene flow direction H3 - > H2; **e** N_e_ = 5 T_3_; data for ${\widehat{f}}_{hom}$, gene flow direction H3 - > H2

Figure 9 shows the difference between the estimated f-statistics from randomly drawn loci and the expected number calculated from the above formulae. The variation of the estimated f-statistics is insensitive to the value of real f. Given the same divergence and introgression times and the same relative population size, ${\widehat{f}}_G$ has a larger margin of error than ${\widehat{f}}_{hom}$, while ${\widehat{f}}_{hom}$ for both gene flow directions have similar error (Fig. 9a, b, c). The variance of the f-statistics also increases with relative population size (Fig. 9b, d, e for ${\widehat{f}}_{hom}$ in H3 - > H2; for ${\widehat{f}}_{hom}$ in H2 - > H3 and ${\widehat{f}}_G$ the result is similar). There is a slight bias for ${\widehat{f}}_{hom}$ when the real f is above 0.2, towards a lower value for H3 - > H2 gene flow and a higher value for H2 - > H3 gene flow (Fig. 9b,c).

**Fig. 10:**
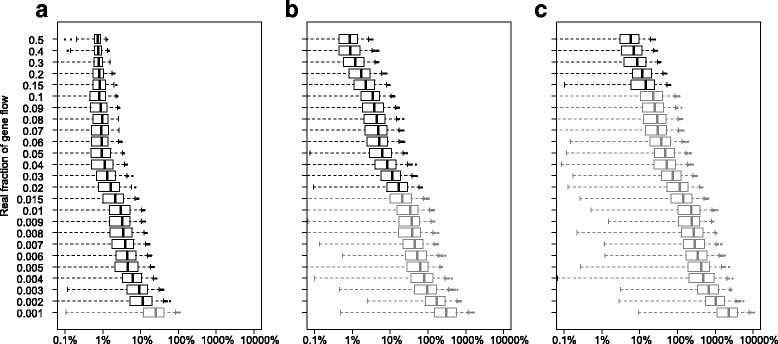
The mean errors (of either direction) of estimated ${\widehat{f}}_G$ (gene flow direction H3 - > H2) compared to the expected value, as the percentage of the expected value. For each scenario and each real f value, 500 bootstrap replicates were calculated. Gray boxes indicate real f values below the sensitivity of the D-statistic in the same scenario (mean of three replicates). In all graphs, the divergence time T_3_ = 1 × 10^5^ generations, T_GF_ = 0.5 T_2_ = 0.25 T_3_. **a** N_e_ = 0.2 T_3_; **b** N_e_ = T_3_; **c** N_e_ = 5 T_3_

However, the expected value of the f-statistic is smaller when the real f is smaller, which means the *relative* error can be large in such cases (Fig. 10). Although in extreme cases with large relative population size and low real f the mean error can be over 10 times the expected value (Fig. 10c), such gene flow events lie outside of the D-statistic’s sensitivity and would not be qualitatively detected at the first place. Generally, the f-statistics can be estimated within ±20% for gene flow events that can be detected, given that population size and divergence and introgression times are known.

**Fig. 11:**
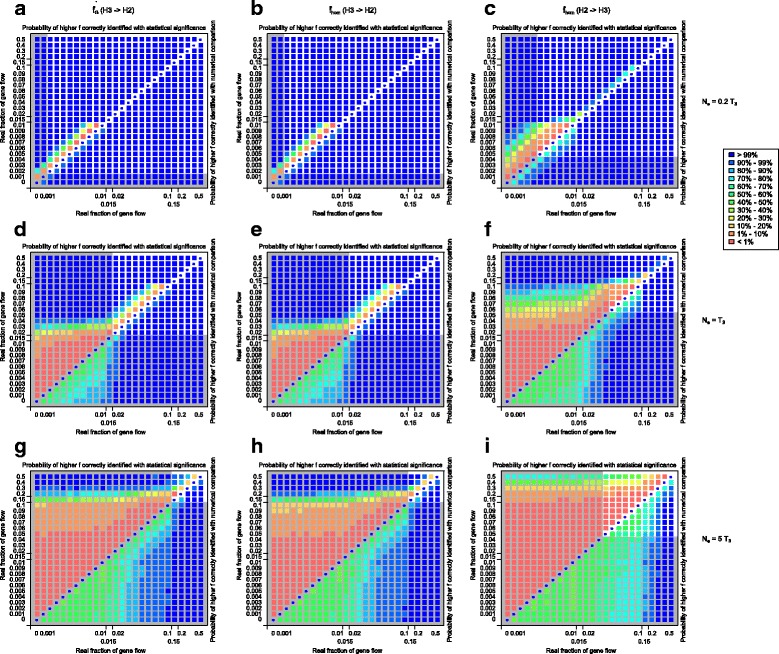
Pairwise comparison between the f-statistics in the same scenario. Each box represents 500 × 500 = 250,000 data points. Upper-left triangle: probability of the higher real f value result in a *statistically* higher f-statistic, based on comparison of confidence intervals. Lower-right triangle: probability of the higher real f value result in a *numerically* higher f-statistic. Diagonal line (circles): probability of *no* statistically significant difference given the *same* real f values. Gray-shaded areas indicate real f values below the sensitivity of the D-statistic in the same scenario (mean of three replicates). In all graphs, the divergence time T_3_ = 1 × 10^5^ generations, T_GF_ = 0.5 T_2_ = 0.25 T_3_. **a**, **b**, **c** N_e_ = 0.2 T_3_; **d**, **e**, **f** N_e_ = T_3_; **g**, **h**, **i** N_e_ = 5 T_3_. **a**, **d**, **g** ${\widehat{f}}_G$, gene flow direction H3 - > H2; **b**, **e**, **h** ${\widehat{f}}_{hom}$, gene flow direction H3 - > H2; **c**, **f**, **i** ${\widehat{f}}_{hom}$, gene flow direction H2 - > H3

Pairwise comparisons showed similar trends (Fig. 11). When the real f are equal, almost all (>99%) of comparisons showed no significant difference regardless of demographic scenario, indicating that the false negative rate of f-statistic comparison is very low (circles on the diagonals).

Relative population size is the main factor in determining whether comparisons between two f-statistics correctly identify the relationship of the real f, either by numerical comparison or statistical significance. Two f values both above the sensitivity of the D-statistic can be confidently correctly compared by numerical f-statistic comparison, even if statistical significance can be lacking with small differences between the f values. While ${\widehat{f}}_G$ (Fig. 11a, d, g) and ${\widehat{f}}_{hom}$ (Fig. 11b, e, h) for H3 - > H2 gene flow shows the same level of robustness, ${\widehat{f}}_{hom}$ for H2 - > H3 (Fig. 11c, f, i) is clearly more difficult to estimate and to compare, under the same divergence and demographic parameters. This is consistent with our results in previous sections where the D-statistic is less sensitive to H2 - > H3 gene flow. In extreme cases with large relative population sizes (Fig. 11i), f values between 0.15 and 0.5 were rarely distinguishable with statistical significance.

The effects of relative divergence and introgression times on robustness of the f-statistics is also similar to that on sensitivity of the D-statistic (data not shown). A larger range of real f values are undistinguishable significantly in datasets with older H1-H2 divergence and introgression, for both ${\widehat{f}}_G$ and ${\widehat{f}}_{hom}$ in the gene flow direction H3 - > H2. On the other hand, for the direction H2 - > H3 the situation is more complicated, with recent divergence causing the most uncertainty and intermediate divergence causing the least.

### Diploid data

In studies based on biological samples, the final genomic sequence is often the consensus of two copies from a diploid organism. The two genome copies from the gene flow recipient population may have different sets of introgressed loci, but our model does not allow explicit estimation of how much the overlap may be. Instead, we simulated two extreme conditions: the two genome copies have either the exact same set of loci affected by gene flow, or independently drawn sets of loci. A realistic expectation lies between these two conditions.

**Fig. 12:**
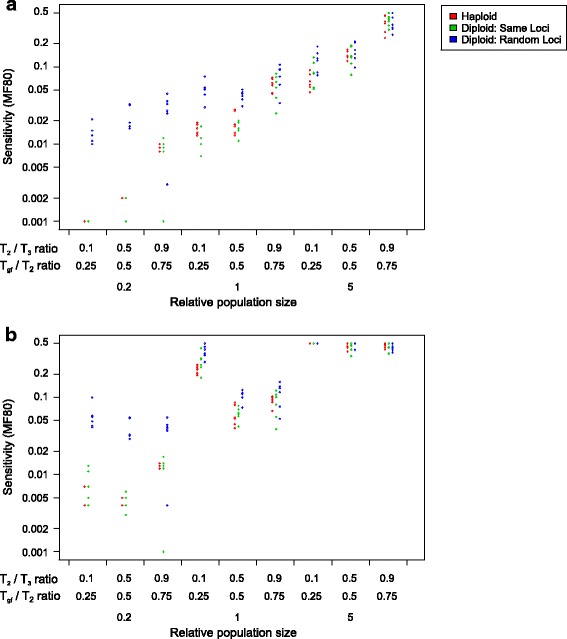
Comparison of sensitivity of the D-statistic in haploid and diploid data. “Same loci” indicates two genome copies share the same set of introgrossed loci, while “random loci” indicates two genome copies have independently drawn sets of introgressed loci. The direction of gene flow is **a** H3 - > H2; **b** H2 - > H3

We found that while the effect of ploidy on the sensitivity (as measured by MF80) of the analysis is not significant (*p* = 0.580 for H3 - > H2, and *p* = 0.537 for H2 - > H3 gene flow), it is significant for H3 - > H2 (*p* = 0.002) as well as for H2 - > H3 (*p* = 0.035) when MF80 is log-transformed. Figure 12 shows the detailed effects of parameters and ploidy on sensitivity. It is evident that diploid data with independent sets of introgressed loci (blue dots) showed considerably increased MF80 values, but only under low relative population size. For datasets with a relative population size of 0.2 (lowest), the sensitivity increased from lower than 0.01 to between 0.01 and 0.05, reaching 0.1 in some cases of H2 - > H3 gene flow. Note that this does not affect datasets with the same set of introgressed loci in both genome copies (green dots).

## Discussion

### Relative population size as the key factor in parameter space

The D-statistic was invented to analyze gene flow between Neanderthals and anatomically modern humans [14]. While it is suitable for both sequence data and population-wide allele frequency data [15], the D-statistic is more often being used on sequences collected from closely-related taxa. Our research explored the parameter space of the phylogeny and demographics of the studied taxa, and determined how these parameters affect the sensitivity of the D-statistic. Sensitivity is described with MF80, the minimal fraction of genome affected by gene flow to produce significant D-statistics in 80% of permutations. An MF80 of 0.01 means that the D-statistic can detect a 1% gene flow.

Our study marks one of the first attempts to explore the parameter space in which the D-statistic is reasonably sensitive. We have shown that the relative population size, which is the ratio of population size and divergence time in generations, is the most important factor on the sensitivity of the D-statistic. Indeed, the “coalescence time unit” as a measure of branch length is the reciprocal of the relative population size, and the probability of gene tree differs from the species tree in a three-species tree is (2/3)e^-t^ where t is the number of coalescence time units [28, 29]. Similarly, the proportion T_2_/T_3_ and T_gf_/T_3_ affects sensitivity through the lengths of branches separating the divergence and gene flow events. Branches short in coalescent time units are likely to produce ILS which add to both ABBA and BABA counts, diluting information for the D-statistic [1]. Our usage of relative population size describes how large the population size is compared to the entire history of the (H1, H2, H3) complex, in contrast to the coalescent time unit, which is used to measure the length of individual branches. In case of changing population sizes, the harmonic mean is commonly used [41]; future research may focus on obtaining a mean population size of multiple diverged populations or species.

Sequence divergence, or genetic distance is not a crucial factor on the D-statistic’s sensitivity alone, at least within a 0.2% to 20% range. The D-statistic analysis has been applied to biological questions with sequence divergences as low as 0.3% [14] and as high as about 5% [26, 27]. The analyses show that within reasonable range, sequence divergence is only a minor concern. A caveat, however, exists in the form of non-substitution mutations. Long-term evolution can accumulate mutations such as insertions, deletions and duplications in the genomes, causing incorrect mapping and alignment, which may affect the gene flow analysis by aligning non-homologous sites and producing artefactual ABBA or BABA sites. Therefore, high-coverage genomes and good alignment tools are essential for such studies.

We scaled both branch lengths and population sizes with the reciprocal of the substitution/mutation rate μ, as it does not affect gene trees (branch lengths measured in substitutions per nucleotide) in a neutral model. This allows the interpretation of our results to be applicable to diverse organisms; humans have a rate of 1.3 to 2.5 × 10^−8^ mutations per nucleotide per generation [42, 43], while in *Heliconius* butterflies the rate is approximately 2 to 3 × 10^−9^ mutations per nucleotide per generation [18, 44, 45]. The relative population size, being a ratio of two parameters both scaled with 1/μ, is completely independent from mutation rate in our model.

In the Neanderthal-human analysis, the relative population size is about 0.5 assuming Ne = 10,000 and T_3_ = 20,000 generations [14]. For the *Heliconius* butterflies, Ne = 0.5 to 2 million and T_3_ = 8,000,000 generations give a relative population size of 0.06 to 0.25 [46]. A study on gibbons gave Ne = circa 100,000, generation time of ten years and T_3_ of circa 5 million years, so the relative population size is 0.2 [47]. These values all fall into a reasonable range in our study. In a study of gene flow among equids, Ne = 200,000, generation time of eight years and divergence time at some 2 million years, giving a relative population size of 0.8 [17]. While this is higher than the previously mentioned studies, strong gene flow over 10% of the genome can still be detected by the D-statistic analyses. Finally, in a study on dogs and wolves, the ancestral Ne was estimated at 35000, generation time of three years and a dog-wolf divergence of circa 15,000 years, resulting in a relative population size of 7 [48]; however, bottlenecks during domestication could have reduced the actual effective population size and consequently incomplete lineage sorting. As a rule, when the relative population size is 0.5 or less, the D-statistic appears to be reliable. On groups with higher population sizes, alternative methods may be required to correctly identify inter-population or inter-species hybridization, using multiple samples within each population. However, studies that find all negative results for the D-statistic may choose not to include these findings in the published manuscript.

### Effects (or lack of effects) of outgroup distance and loci number

Other parameters, such as outgroup distance and loci number, were also explored on their effect on the statistical analysis. A distant outgroup has been shown to, for example, reduce accuracy of phylogenetic rooting [49]. Counterintuitively, the distance between the outgroup and the ingroups within reasonable range seems not to be relevant to the sensitivity of the D-statistic as well as the errors of the f-statistics. The determining of ancestral allele in ABBA or BABA sites is based on the outgroup. It might be expected that a distant outgroup would cause more false positives and negatives in identifying such sites due to multiple substitutions (randomization), reducing the efficiency of the D-statistic to detect gene flow. Simulating multiple substitution is possible with the program INDELible, because it evolves DNA sequences with each mutation being independently assigned. While the concern about alignment and mapping artifacts with a very distant outgroup still exist, it is a reassurance that the D-statistic analysis can be used even when a closely related species as an outgroup cannot be found.

The D-statistic is also shown to be more sensitive in a large number of smaller loci, given a constant size of analyzed genome. The most likely reason is a lower sampling error; even without gene flow, a single locus can favor ABBA or BABA sites due to incomplete lineage sorting. A larger number of loci means that the number ABBA-favoring and BABA-favoring loci are more similar to each other.

While we did not use a complex recombination model, loci number can be seen as a proxy of recombination rate. A higher recombination rate will break up linkage more often, which leads to an increase of locus number. Based on our results, when other conditions are similar, taxa with higher recombination rates are more sensitive to the D-statistic. Furthermore, given a constant rate of recombination, longer divergence time between taxa means more recombination events, thus increasing sensitivity because of reduced locus size; as we have shown in a previous section, genetic distance alone does not reduce sensitivity. Our main simulation scheme provides 20 kb loci, while the shortest loci are 10 kb. Further reduction of length (thus increasing the number of independent loci) is constrained by computation time limitations. In biological datasets, if the loci are even shorter, one can expect even better sensitivity of the D-statistic compared with our results. In the future, backward simulations based on coalescence algorithms, such as msprime [50], can be employed to further pinpoint the effects of locus size and recombination rate.

### Direction of gene flow

The D-statistic was developed to detect gene flow in both directions, i.e. H2 - > H3 and H3 - > H2. In the studies on Neanderthal and human genomes, Neanderthals are extinct and multiple non-African modern human populations were used, therefore it is reasonable to claim that the direction is from Neanderthal to modern humans [14, 15]. However, with studies where all sampled taxa are extant and only one sample is available for each of the four taxa, the D-statistic alone cannot determine the direction of gene flow. A five-taxon statistic known as D-FOIL is able to determine direction of gene flow in some situations [51].

We have observed that, other parameters being identical, H3 - > H2 gene flow is easier to detect than H2 - > H3 gene flow. A mutation that produces ABBA sites under H3 - > H2 gene flow must occur after H3 diverged from H1/H2 (T_3_) and before the gene flow (T_GF_); but such mutation under a H2 - > H3 gene flow must occur after H2 diverged from H1 (T_2_) and before the gene flow (T_GF_). The former timespan is longer than the latter under the same demographic scenario; therefore, when the f is equal, H3 - > H2 gene flow produces more ABBA sites than H2 - > H3, making it easier to be detected with the D-statistic.

There is also an interesting finding regarding the direction of gene flow: more recent divergence between H1 and H2 hinders the detection of H2 - > H3 gene flow but helps the detection of H3 - > H2 gene flow. The former can be explained by that the timespan between T_2_ and T_gf_, required for ABBA sites under H2 - > H3, is reduced when T_2_ is smaller. The latter may be explained by that more recent H1-H2 divergence means their (pre-introgression) sequences are more similar, providing a clearer background for the introgressed sites to be detected.

### False positives

False positives are also a potential problem for the D-statistic. While only one simulated dataset in a total of 789 has a |Z| > 3, 45 out of the these datasets in the main simulation scheme have |Z| > 1.96, which would be significant had we set the significance level to be *p* < 0.05; this is consistent with the proportion of such datasets (5.7%). There are two main sources of false positives in the D-statistic. One is loci sampling error. ILS produce gene trees that group H1 and H3 together (favoring BABA sites) at the same rate as gene trees that group H2 and H3 together (favoring ABBA sites), which theoretically cancel out with each other. However, as the number of sampled loci is finite, the two types of gene trees may have different frequencies by chance, causing BABA and ABBA site number to be unbalanced. The other source of error is that the sequence from H1 being more or less distant than H2 from the H1-H2 common ancestor. This can be caused by a different evolutionary rate or sequencing error, the latter of which have been analytically tested by [15] for one specific set of parameter values.

To date, there is no simulation-based study on false positives of the D-statistic from either source, nor analysis on the interaction of them and other factors such as population size. This is possibly due to the fact that specificity tests, i.e. tests of false positive rates, require independent replicate datasets on which the D-statistic would be calculated under no gene flow. If all 135 parameter combinations of our main data scheme are simulated 500 times in parallel, the total running time would be estimated as 4.5 years. Future studies may focus on finding methods of permutation and subsampling so that independent or almost independent datasets can be generated within limited computation power, on which false positives can be studied.

### Usage of the f-statistics

Our result showed that both ${\widehat{f}}_G$ and ${\widehat{f}}_{hom}$are largely linearly correlated with the real fraction of gene flow, f, but the usage of them to estimate f is hindered by parameters that are often unknown. These include the direction of gene flow and the T_gf_/T_3_ ratio - the relative chronologic placement of the gene flow event. Sampling error due to finite number of loci can also introduce uncertainty, particularly when the fraction of gene flow is small. We conclude that the f-statistics cannot be used to reliably estimate the true fraction of gene flow without polymorphism data from a larger number of individuals in each population, *or* reliable estimates of divergence and introgression times as well as population sizes.

The linearity between the real f and f-statistics can be exploited to compare the extent of introgression between different genomic regions with the same set of taxa, or taxa that have similar divergence times and population sizes. Our results showed that within the sensitivity of the D-statistic (which means that gene flow events can be detected at the first place), the random error of the f-statistics are moderate; a ± 20% error must be taken into account. However, a higher real f may not be statistically significant especially when the f values are just above the D-statistic’s sensitivity. It is common for a higher real f to always report a numerically higher f-statistic but seldom statistically significant. Therefore, when comparing the f-statistics resulting from multiple tests under the same evolutionary scenario, it is preferred to choose numerical comparison under difficult conditions (large relative population size, early divergence/introgression and small difference between gene flow fractions), and to choose statistical comparison otherwise.

A few other f-statistic applications exist, but some of them require more sampled lineages than three ingroups and one outgroup; an example is f_4_-ratio estimation [31]. Others require population data, in which the allele frequencies from different populations are used, such as f_2_ and f_3_ statistics [38] as well as ${\widehat{f}}_d$ [22]. Here we focused on the situation that only one or two sequences are sampled from each taxon, and four taxa (three ingroups and one outgroups) are sampled; our conclusion is that, in such cases, ${\widehat{f}}_G$ and ${\widehat{f}}_{hom}$ are not very reliable estimator of actual fraction of gene flow, f, except in special cases when population is very small and the time of gene flow is known.

### Diploid data

We have shown that the sensitivity from diploid sequences where the two genome copies have different introgressed loci can be worse than haploid sequences or diploid sequences with the same set of introgressed loci, especially when the sensitivity is good at the first place (low relative population size). It is expected that, the more recent the gene flow happens, the more likely that two genome copies would have different set of introgressed loci. The reason for this is that alleles from recent migrants may not be fixed or lost yet, and segregate in the recipient population leading to heterozygous sites. When the heterozygous sites were removed in constructing a consensus sequence, valuable data to estimate gene flow were also lost. Therefore, our suggestion is that haploid sequence (acquired by sequencing haplotypes, or randomly assign one nucleotide for heterozygote sites) instead of consensus sequence from diploid samples should be used, when gene flow is recent *and* relative population size is small; otherwise consensus from diploid data is sufficient.

Still, our model does not explicitly predict how many introgressed sites are shared by the two genome copies, but analyzed two extreme situations and interpolated. In the future, it is possible that a more sophisticated coalescent-based model can be used to further investigate the effect of diploid data on the D-statistic.

### Potential gene flow from an extinct or unsampled lineage

**Fig. 13:**
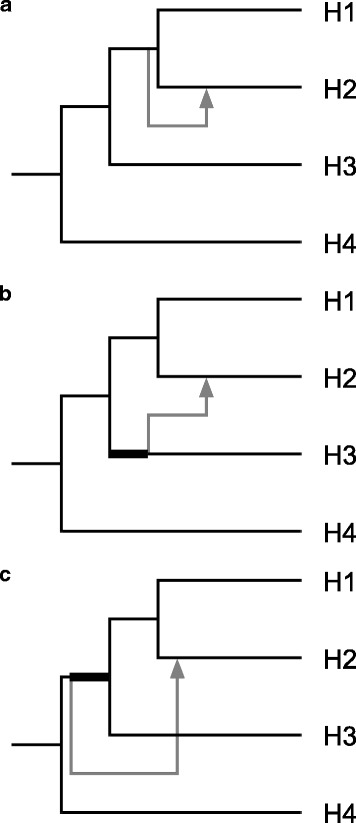
Three possibilities with a “ghost lineage.” A ghost lineage is an extinct or unsampled lineage (in gray), that introgressed into a sampled lineage (H2 in this case). **a** Ghost lineage diverged after H3 did, making it a sister lineage of (H1 + H2). In this case it is impossible to have one mutation produce an ABBA or BABA site pattern. **b** Ghost lineage as a sister lineage of H3. In this case it is similar to gene flow originating from H3; if a mutation occurs in the bolded branch it can produce an ABBA site pattern, which can be interpreted as evidence of gene flow between H2 and H3. **c** Ghost lineage diverged before H3 did. If a mutation occurs in the bold branch it can produce a BABA site pattern, which can be (incorrectly) interpreted as evidence of gene flow between H1 and H3

Another possibility is that the gene flow originates from a lineage that is extinct or unsampled, commonly referred to as a “ghost lineage.” Figure 13 shows three possible origins of such a lineage. When the “ghost” diverged after H3 did (Fig. 13a), the event cannot be detected by the D-statistic, as a single mutation on the tree of an introgressed loci cannot produce either ABBA or BABA patterns. When the “ghost” is a sister species of H3 (Fig. 13b), the situation is identical to gene flow from H3 at the time when H3 and “ghost” diverged; any mutation occurring on the bolded branch can produce a species-tree-discordant site pattern. However, when the ghost diverged *before* H3 did (Fig. 13c), the situation is more complicated. A mutation occurring on the bold branch would have descendants in H1, H2 and H3, and if then the locus in H2 is replaced by a plesiomorphy from the ghost lineage, the site pattern will be “BABA,” just like a gene flow *from H3 to H1*. Durand et al. [15] calculated the expected D-statistic during such a situation, but using only four taxa there is no method to differentiate such a “ghost introgression” from a gene flow from H3. Biologists would need to sample a larger range of species to determine where an introgression lineage come from.

## Conclusion

In this study, we have shown that the D-statistic is more sensitive in detecting gene flow events when a) the population size to divergence ratio is small, b) gene flow is recent, and c) in the direction of H3 - > H2 (compared to H2 - > H3), and d) the data contains larger number of independent loci. On the other hand, the D-statistic is less sensitive to different levels of sequence divergence among ingroups or between ingroups and the outgroup. We have established the reliability of the D-statistic under a large range of parameter space. The f-statistics, while linearly correlated with the fraction of genome affected by gene flow, is not reliable for most applications with individual (rather than population) samples, because its dependence on too many parameters, such as the time of gene flow event which can be difficult to accurately estimate; however it can be used to compare amount of introgression in the same demographic scenario. We have established that, as a rule of thumb, under a population size that equals or less than half of the number of generations since divergence of all tested species, the D-statistic is a sensitive method to detect gene flow.

## Additional files

Additional file 1: Sensitivity and input parameters (continued). Description: The relationship of sensitivity as measured with MF80, the minimal fraction of gene flow that produces over 80% significant D-statistics, and various input parameters: A. the ratio of divergence times, T_2_ and T_3_; B. the ratio of time of gene flow (T_gf_) and T_2_. Red points represent gene flow from H3 to H2, and green points represent gene flow from H2 to H3. (PDF 150 kb)

Additional file 2: Detailed results from each dataset. Description: Input parameters, sensitivity, significance information and linear regression of the f-statistics, from all datasets in three simulation schemes. (XLSX 214 kb)
