# Supplementary figures and images for: Gene flow analysis method, the D-statistic, is robust in a wide parameter space
Source: BMC Bioinformatics. 2018 Jan 8;19:10. doi: 10.1186/s12859-017-2002-4 (PMC5759368; doi:10.1186/s12859-017-2002-4)

A

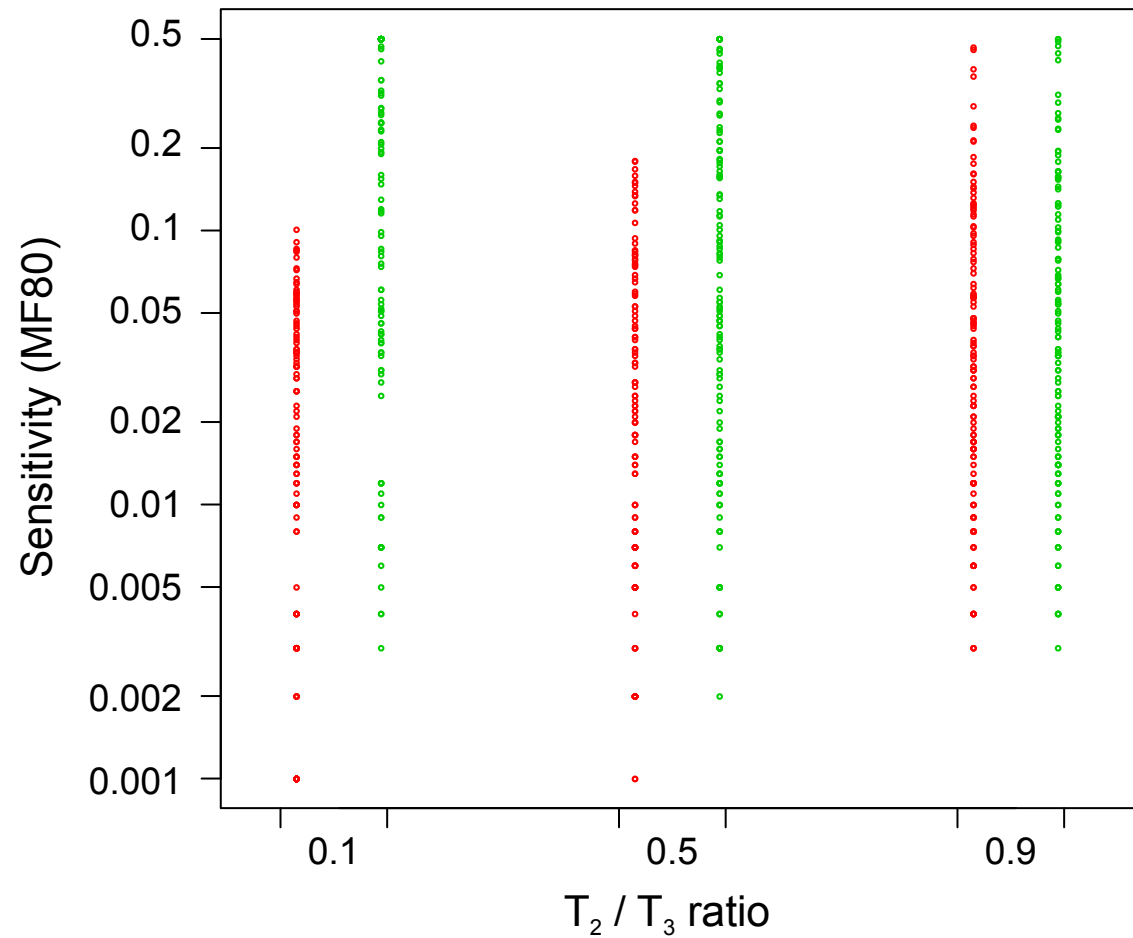

B

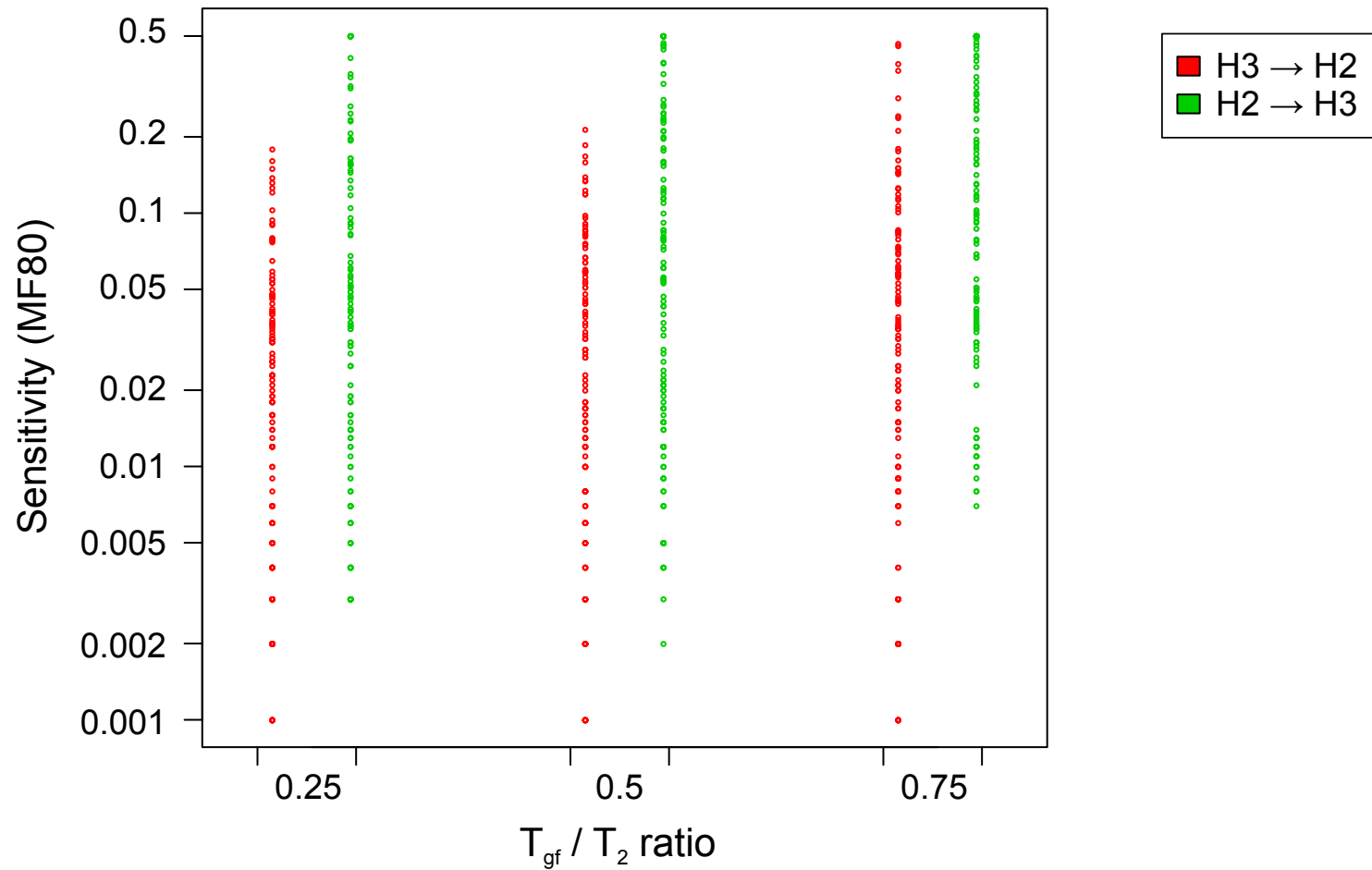

Supplement: Supplementary file 1 — Sensitivity and input parameters (continued). Description: The relationship of sensitivity as measured with MF80, the minimal fraction of gene flow that produces over 80% significant D-statistics, and various input parameters: A. the ratio of divergence times, T2 and T3; B. the ratio of time of gene flow (Tgf) and T2. Red points represent gene flow from H3 to H2, and green points represent gene flow from H2 to H3. (PDF 150 kb) [file 12859_2017_2002_MOESM1_ESM.pdf]
